# Validating the 2022 WHO classification in thyroid carcinomas: Prospective evidence for predicting iodine resistance

**DOI:** 10.1111/his.70148

**Published:** 2026-03-28

**Authors:** Elise André, Laurine Mebarki, Mirela Ilie, Fabien Subtil, Claire Bournaud, Françoise Borson‐Chazot, Françoise Descotes, Jean‐Christophe Lifante, Mireille Bertholon‐Grégoire, Hélène Lasolle, Myriam Decaussin‐Petrucci

**Affiliations:** ^1^ Pathology Department Centre Hospitalier Lyon Sud, Hospices Civils de Lyon Pierre Bénite France; ^2^ Fédération d'Endocrinologie, Groupement Hospitalier Est, Hospices Civils de Lyon Bron France; ^3^ Cancer Research Center of Lyon, INSERM1052 CNRS5286, Lyon 1 University Villeurbanne France; ^4^ Biostatistics Department Lyon 1 University, Hospices Civils de Lyon, CNRS, Laboratoire de Biométrie et Biologie Evolutive IMR 5558 Villeurbanne France; ^5^ Department of Endocrinology and Diabetology CH de Roanne Roanne France; ^6^ Lyon 1 University Villeurbanne France; ^7^ Biochemistry and molecular biology department Centre Hospitalier Lyon Sud, Hospices Civils de Lyon Pierre Bénite France; ^8^ Endocrine Surgery Department Centre Hospitalier Lyon Sud, Hospices Civils de Lyon Pierre Bénite France; ^9^ Nuclear Medecine Department Groupement Hospitalier Est, Hospices Civils de Lyon Bron France

**Keywords:** high‐grade differentiated carcinomas, high‐grade thyroid carcinomas, radioactive iodine refractory, thyroid carcinomas, WHO classification

## Abstract

**Objective:**

To validate whether the 2022 WHO classification improves the prediction of radioactive iodine refractory (RAIR) disease in advanced‐stage thyroid carcinomas and to identify associated histological predictive factors.

**Methods and results:**

A prospective cohort of 253 patients with pT3, pT4, or M1 thyroid carcinomas (TNM 2010), who underwent surgery between 2009 and 2018 and received at least one radioactive iodine dose, was analysed. Histological slides were reviewed using the 2022 WHO criteria, and clinical data were collected. Cox survival analyses and ROC curve evaluations were performed to identify factors associated with RAIR progression and assess the predictive performance of the new classification. Reclassification identified 28 low‐risk neoplasms, all with favourable outcomes. Among the remaining 225 malignant cases, 155 were well‐differentiated carcinomas (including 146 papillary carcinomas, 56 of which were aggressive subtypes) and 70 were high‐grade carcinomas (39 high‐grade differentiated and 31 poorly differentiated carcinomas). Forty patients (17.7%) developed RAIR disease. The 2022 WHO classification showed superior predictive performance compared with the 2004 WHO (AUC: 0.81 versus 0.60, *P* < 0.001). Multivariate analysis identified tumour necrosis as independent predictive factor of RAIR disease, regardless of the mitotic index.

**Conclusion:**

The 2022 WHO classification improves RAIR disease prediction, notably by recognizing high‐grade carcinomas as a distinct entity. High‐grade factors, especially tumour necrosis, which emerges as a key predictive factor, should be systematically reported in thyroid carcinoma pathology reports to enhance patient management and follow‐up.

## Graphical abstract

This study assessed the 2022 WHO classification for predicting RAIR disease in thyroid carcinomas, comparing it to older classifications. In a retrospective study of 253 cases, we found the 2022 classification more effective, especially by identifying high‐grade carcinomas and tumour necrosis as critical predictive factors.

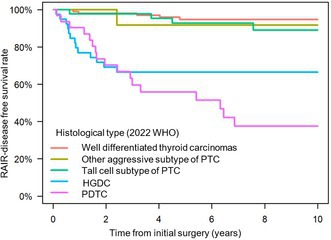

**Abbreviations**
AUC: area under curve
HGDC: high‐grade differentiated carcinomas
HR: hazard ratio
PDTC: poorly differentiated thyroid carcinomas
PTC: papillary thyroid carcinomas
RAI: radioactive iodine
RAIR: radioactive iodine refractory

## Introduction

Thyroid carcinomas are predominantly well‐differentiated, representing more than 90% of cases,^1^ with a majority of papillary thyroid carcinomas (PTC). These tumours generally have an excellent prognosis, with a 5‐year survival rate of 95%.^2^ However, approximately 5%–10% develop distant metastases, and two‐thirds of them become refractory to radioactive iodine (RAIR), the main therapeutic option for differentiated thyroid carcinomas.^3^ RAIR disease is the primary factor contributing to disease‐specific mortality, with a 10‐year survival rate of less than 10%.^3^ Identifying patients at risk of iodine refractory progression remains a major challenge in the management of thyroid cancer.

Histopathological classifications have evolved to improve risk stratification. The 2004 WHO classification^4^ introduced poorly differentiated thyroid carcinomas (PDTC), a distinct group of aggressive tumours. However, some well‐differentiated carcinomas also show poor outcomes similar to PDTC.^4^ The challenge of identifying these aggressive tumours early led to the search for additional histopathological criteria to improve classification and prognostication. In this context, some studies proposed scoring systems based on tumour necrosis and mitotic activity.^5^ These findings were incorporated into the 2022 WHO classification,^6^ which introduced the concept of high‐grade differentiated carcinomas (HGDC). This new category includes PDTC and tumours that maintain the architectural characteristics of papillary, oncocytic, or follicular carcinomas but present at least one of the following two criteria: tumour necrosis and/or a mitotic index greater than 5/2 mm^2^ in hotspot areas. By grouping HGDC and PDTC under the broader category of high‐grade follicular cell‐derived non‐anaplastic thyroid carcinoma, this classification aims to refine prognostic stratification by identifying tumours with a higher risk of aggressive clinical behaviour.

Given the prognostic implications of these new classifications, reassessing their clinical relevance is essential. The objective of our study was to confirm the better performance of the 2022 WHO classification in predicting the risk of RAIR disease, comparing it with previous classifications and identifying prognostic histological factors associated with iodine resistance. To do so, we reclassified a prospective cohort of 253 thyroid carcinomas operated on between 2009 and 2018, initially classified according to the 2004 WHO classification and 2010 pTNM systems. This analysis aims to provide prospective evidence whether the integration of 2022 WHO classification into routine practice improves the identification of patients requiring adapted therapeutic strategies.

## Materials & Methods

### Study Cohort

A large prospective cohort of patients who underwent thyroid carcinoma surgery and radioactive iodine (RAI) therapy between June 2009 and March 2018 in a tertiary thyroid expert center (Hospices Civils de Lyon, University hospital of Lyon, France) was analysed. Inclusion criteria were: (i) age ≥18 years; (ii) diagnosis of thyroid carcinoma of follicular origin; (iii) pT3, or pT4 or M1 stage at diagnosis according to the 2010 pTNM classification; (iv) treatment with at least one dose of radioactive iodine. Clinical and histological data were recorded using Access software.

We excluded: (i) patients operated outside our institution without histological material; (ii) cases with material not from the primary tumour; (iii) patients with follow‐up <1 year.

The study was approved by ethical commitee (2011‐A00772‐39), and written informed consent was obtained from all patients. Data were handled confidentially following the French Data Protection Authority (CNIL) guidelines.

### Histological Review

Haematoxylin and eosin‐stained slides from selected cases were retrieved from the Hospices Civils de Lyon archives and independently reviewed by a junior pathologist (EA) and a senior expert in thyroid pathology (MDP) member of the thyroid french network ENDOCAN‐TUTHYREF.^7^, ^8^ Pathologists were blinded to clinical data.

Reviewed data were recorded in a new Access database, integrating 2022 WHO and 2017 pTNM classifications alongside prognostic histological criteria.

At the time of initial diagnosis, all the cases were classified according to the 2004 WHO classification. Final diagnoses were assigned according to 2022 WHO, distinguishing non‐invasive follicular thyroid neoplasm with papillary‐like nuclear features (NIFTP), tumours of uncertain malignant potential (UMP‐T), and thyroid carcinoma subtypes.

pTNM staging was reassessed according to the 8th edition (2017).

### Immunohistochemistry and Molecular Analysis

A Ki‐67 proliferation index (MIB‐1 antibody, BenchMark‐ULTRA/VENTANA RocheDiagnostics, Meylan, France) was assessed for cases staged as pT2 or higher.

DNA was isolated from formalin‐fixed paraffin‐embedded tissue blocks of tumour thyroid tissue, using the QIAamp‐DNA‐FFPE tissue kit on a QIAcube instrument (Qiagen, Hilden, Germany). Mutations of *BRAF* exon 15, *NRAS* exon 3, *HRAS* exon 3 were screened using real‐time PCR and fluorescence high‐resolution melting curve analysis on a LightCycler480 instrument (RocheDiagnostics, Vienna, Austria). Positive samples were confirmed by Sanger sequencing.^9^ Mutations of *TERT* promoter were analysed using nested PCR and sequencing as follows: previously described.^10^

### Clinical Follow‐Up

Clinical data included RAIR status (2015 American Thyroid Association), disease‐related mortality, and distant (non‐cervical) metastases. Patients were considered as RAIR if they met one of the following criteria: no RAI uptake in metastatic sites initially or during therapy; or heterogeneous uptake of metastases; or progression within 12 months after RAI despite uptake or progression despite ≥600 mCi cumulative dose.

### Statistical Analysis

Statistical analyses were performed using R software, version 4.0.2. Quantitative variables were described as median (with interquartile range). Categorial variables were presented as counts and pourcentages. Chi‐squared test and Wilcoxon test were used to assess statistical significance for differences among groups. Primary outcome was factors associated to RAIR survival, defined as the time from initial surgery until at least one of the above criteria was met. Kaplan–Meier curves were used to describe RAIR survival in several subgroups. Univariable and multivariable analyses of factors associated with RAIR survival were performed using Cox models. The Bonferonni method was used to correct for multiple testing in univariable and multivariable analyses, adjusted *P*‐values are presented. Association with RAIR survival was quantified by the hazard ratio (HR) with the associated 95% Confidence Interval (95% CI). Multivariable analyses included only variables significant in univariable analysis (*P* < 0.05). For these analyses, NIFTP and UMP‐T were excluded. ROC curves were used to compare the performance of the WHO 2004 versus 2022 classification models in predicting RAIR disease.

## Results

### Demographic and Prognostic Histological Criteria

The clinical and histological characteristics of the cohort are summarized in Table 1. The study included 253 patients, with a female predominance (62.4%). The median age at diagnosis was 51 years (37–75), with a median tumour size of 4.20 cm (2.60–5.50). Extrathyroidal extension was observed in 54.9% of cases, vascular invasion in 43.9% of tumours, and necrosis in 18.2%. High mitotic activity (≥3 mitoses/2 mm^2^) was found in 28.5% of cases, and Ki67 ≥4% in 18.2%.

**Table 1:** demographics and histological characteristics

Characteristics	*N* = 253 (%)
Gender	
Female	158 (62.4%)
Male	95 (37.6%)
Age at diagnosis (years)	
Median (Q1–Q3)	51 (37–75)
Tumour size (cm)	
Median (Q1–Q3)	4.20 (2.60–5.50)
Single or multifocal lesion	
Single	148 (58.5%)
Multifocal	105 (41.5%)
Encapsulated lesion	
No	150 (59.3%)
Yes	103 (40.7%)
Extra‐thyroid extension	
None	114 (45.1%)
Adipose tissue only	102 (40.3%)
Perithyroid muscle (microscopic)	1 (0.4%)
Perithyroid muscle (gross invasion)	22 (8.7%)
Adjacents organs	14 (5.5%)
Vascular invasion	
None	142 (56.1%)
Presence	111 (43.9%)
Necrosis	
None	207 (81.8%)
Presence	46 (18.2%)
Mitotic index (/2 mm^2^)	
<3	186 (73.5%)
3 or 4	24 (9.5%)
5 or more	43 (17.0%)
Proliferation index (Ki67)	
<4%	159 (63.8%)
4% or more	46 (18.2%)
Not available	48 (19.0%)
Perineural invasion	
None	242 (95.6%)
Presence	11 (4.4%)
Lymph node metastasis	
None	32 (12.6%)
Presence	94 (37.2%)
No lymph node removal	127 (50.2%)
RAIR disease	
No	213 (84.2%)
Yes	40 (16.8%)
Disease‐related deaths	17 (6.7%)

### pTNM Staging Reclassification

A total of 28 nodules (11%) were reclassified as low‐risk neoplasms and no longer required pTNM staging. Comparison of pTNM stages (UICC 7th and 8th editions) of the 225 cancers showed that staging remained unchanged for 65% of cases. However, 79 patients (35%) were downgraded to lower stages (pT1a, pT1b, or pT2). Downstaging between editions was exclusively related to the exclusion of microscopic extrathyroidal extension from the T3 category in the 8th edition, leading to reclassification of these tumours as T1 or T2, according to tumour size.

### Initial Histological Diagnosis and Review

The initial 2004 WHO histological classification and its reclassification according to 2022 WHO are presented in Table 2.

**Table 2:** comparison of initial and review histological data, according to WHO 2004 and 2022 classification respectively

Histological features	2004 WHO	2022 WHO
	Initial histological examination *N* (%)	Histological review *N* (%)
NIFTP	‐	22 (8.7%)
UMP‐T	‐	6 (2.4%)
Well‐differentiated carcinomas	228 (90.1%)	155 (61.3%)
Papillary carcinoma	209 (91.7%)	146 (94.2%)
Classic	85 (40.7%)	46 (31.5%)
Tall cell	41 (19.6%)	44 (30.1%)
Infiltrative follicular	70 (33.5%)	10 (6.8%)
Invasive encapsulated follicular		18 (12.3%)
Encapsulated classic	‐	9 (6.2%)
Solid/trabecular	11 (5.3%)	9 (6.2%)
Whartin‐like	‐	5 (3.4%)
Diffuse sclerosing	2 (0.9%)	2 (1.4%)
Columnar cell	‐	1 (0.7%)
Oncocytic	‐	2 (1.4%)
Follicular carcinoma	19 (8.3%)	3 (1.9%)
Encapsulated angioinvasive	6 (31.6%)	2 (66.7%)
Minimally invasive (capsular invasion only)	8 (42.1%)	1 (33.3%)
Widely invasive	5 (26.3%)	‐
Oncocytic carcinoma	‐	6 (3.9%)
Encapsulated angioinvasive		2 (33.3%)
Minimally invasive (capsular invasion only)		3 (50.0%)
Widely invasive		1 (16.7%)
Differentiated high‐grade carcinomas	‐	39 (15.4%)
Papillary carcinoma		32 (82.0%)
Infiltrative follicular		2 (6.3%)
Invasive encapsulated follicular		11 (34.4%)
Tall cell		9 (28.1%)
Classic		5 (15.6%)
Solid/trabecular		3 (9.4%)
Columnar cell		1 (3.1%)
Oncocytic		1 (3.1%)
Follicular carcinoma		4 (10.3%)
Encapsulated angioinvasive		2 (50.0%)
Minimally invasive (capsular invasion only)		2 (50.0%)
Widely invasive		‐
Oncocytic carcinoma		3 (7.7%)
Encapsulated angioinvasive		1 (33.3%)
Minimally invasive (capsular invasion only)		1 (33.3%)
Widely invasive		1 (33.3%)
Poorly differentiated carcinomas	25 (9.9%)	31 (12.2%)

**Figure 1:**
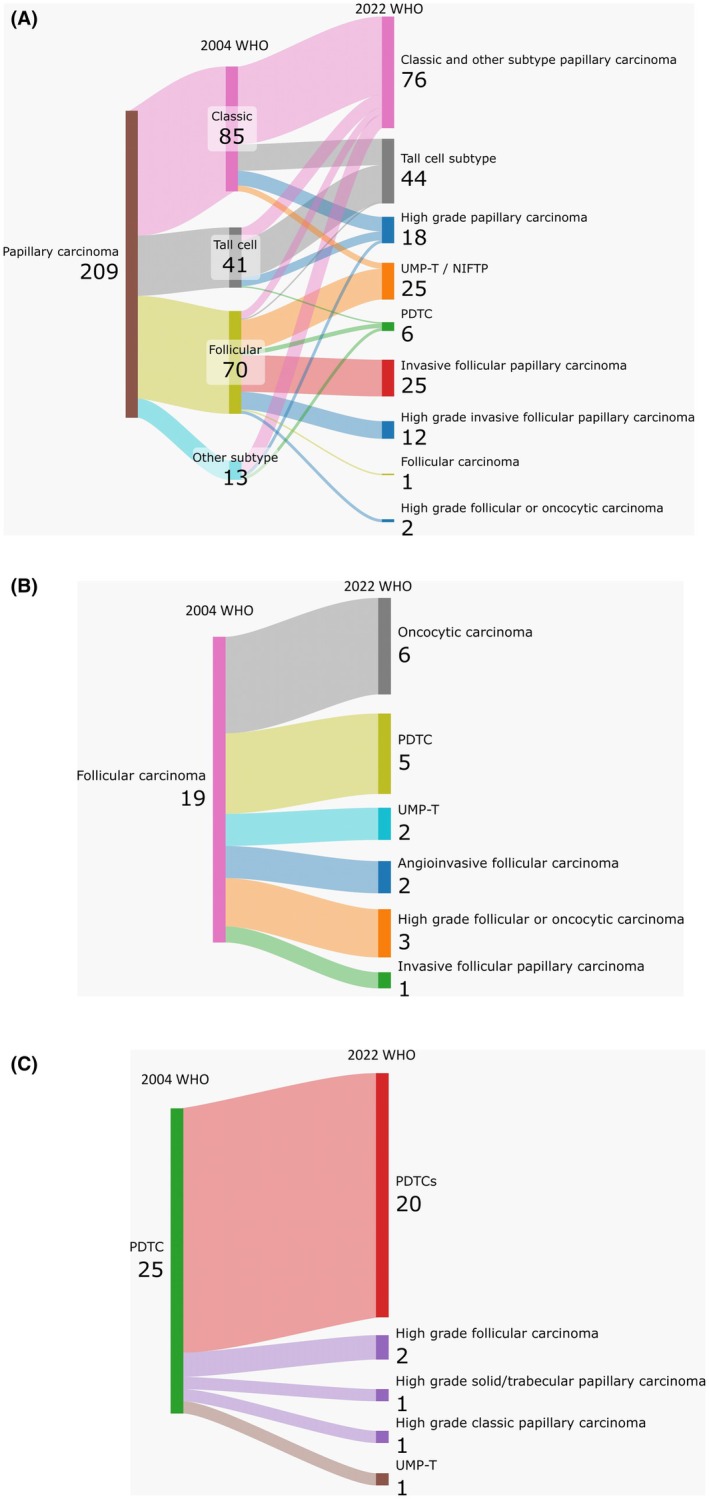
Reclassification from the 2004 WHO to the 2022 WHO classification of: **(A)** papillary thyroid carcinoma; **(B)** follicular thyroid carcinoma; **(C)** poorly differentiated thyroid carcinomas. NIFTP, non‐invasive follicular thyroid neoplasm with papillary‐like nuclear features; PDTC, poorly differentiated thyroid carcinoma; UMP‐T, uncertain malignant potential tumour.

Figure 1 illustrates the distribution of histological types of 2004 WHO and their reassignment according to the 2022 WHO criteria.

Twenty‐eight (11%) tumours were reclassified as low‐risk neoplasms (NIFTP and UMP‐T). None of these patients developed recurrence, metastasis, or RAIR disease after a minimum follow‐up of 3 years.

Initially, according to the 2004 WHO classification, 90% of cases were classified as well‐differentiated carcinomas, with PTC (83%) being the predominant type. Aggressive subtypes (tall cell, diffuse sclerosing, solid/trabecular) represented 26% of PTC. Poorly differentiated carcinomas accounted for 10% of cases, and follicular carcinomas for 8%.

Following the 2022 WHO criteria, reclassification resulted in: 146 PTC (58%), 6 oncocytic carcinomas (2%), 3 follicular carcinomas (1%), 70 high‐grade carcinomas (28%), and 28 low‐risk neoplasms (11%).

Among PTC, 62% belonged to common subtypes, and 38% to aggressive ones, with tall cell subtype being the most frequent (30%).

High‐grade carcinomas represented 28% of the cohort, subdivided into HGDC (*n* = 39; 15%) and PDTC (*n* = 31; 12%). HGDC were classified based on the presence of tumour necrosis (*n* = 13; 33%), a mitotic count ≥5 mitoses/2 mm^2^ (*n* = 18; 46%), or both criteria combined (*n* = 8; 21%).

Among HGDC, 32 (82%) were PTC, including 13 (41%) with aggressive subtypes (9 tall cell, 1 columnar cell, 3 trabecular/solid).

Univariate analysis showed that high‐grade carcinomas were significantly associated with male gender (*P* = 0.002), older age at diagnosis (*P* < 0.001), larger tumour size (*P* < 0.001), more frequent vascular invasion (*P* < 0.001), extrathyroidal extension (*P* = 0.015), and M1 stage at diagnosis (*P* = 0.004). There was no significant association with lymph node metastasis (*P* = 0.6).

### Molecular Profile

Molecular analysis performed for 225 cases carcinomas found (Table 3): 89 (39.5%) *BRAF V600E* mutations, 33 (14.6%) *RAS‐like* mutations (19 *NRAS*, 13 *HRAS*, 1 *BRAF K601E*) and 98 (43.5%) wild‐type. In univariate analysis, a wild‐type *BRAF* profile was significantly associated with high‐grade carcinomas (*P* < 0.001), whereas no statistically significant association was found between high‐grade carcinoma and *RAS*‐like mutations (*P* = 0.09).

**Table 3:** *BRAF*‐like, *RAS*‐like and *TERT* promoter mutations according to histological types

Histological type	Wild‐type	*BRAF‐like* mutation	*RAS‐like* mutation	*TERT* promoter mutation	Data not available^a^
Well‐differentiated carcinomas					
Papillary carcinoma (*N* = 146)	50	76	17	22	3
Follicular or oncocytic carcinoma (*N* = 9)	6	0	3	3	‐
Differentiated high‐grade carcinomas					
Papillary carcinoma (*N* = 32)	15	11	4	13	2
Follicular or oncocytic carcinoma (*N* = 7)	4	0	3	1	‐
Poorly differentiated carcinomas (*N* = 31)	23	1	6	10	1
Total	98	88	33	49	6

^a^ Molecular biology not performed or poor‐quality DNA.

*TERT* promoter mutation was identified in 49 cases (21.8%) with a statistically significant association with high‐grade carcinomas in univariate analysis (*P* = 0.003).

### Prognostic Histological Criteria Associated with RAIR Disease

The median follow‐up was 8 years. Among the 225 carcinomas, 40 (17.8%) developed RAIR disease, with a mean time to onset of 2.3 years.

**Figure 2:**
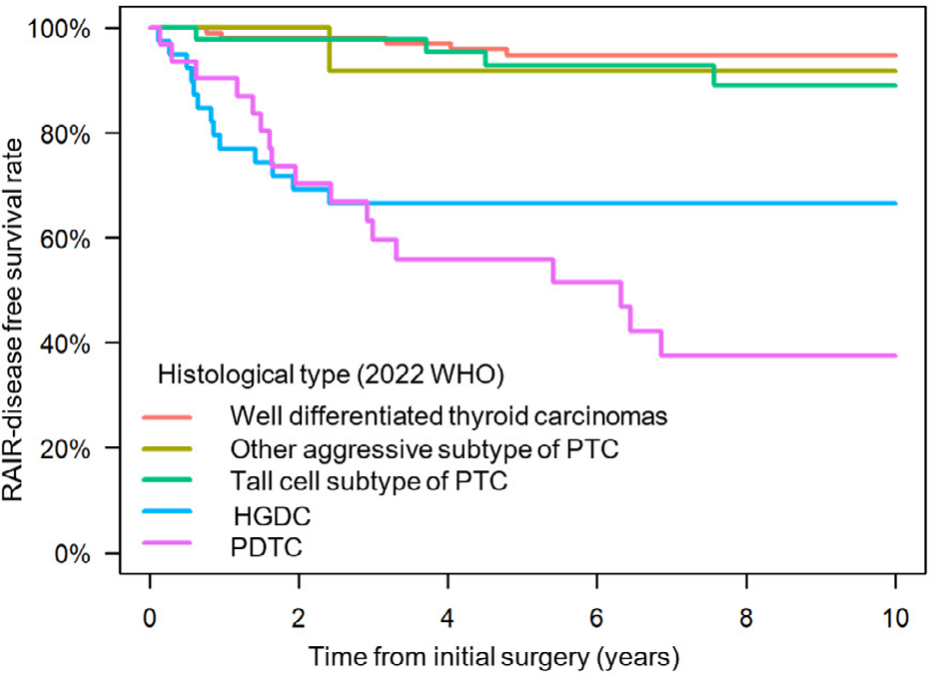
RAIR disease‐free survival curves according to histological type. HGDC, high‐grade differentiated carcinoma; PDTC, poorly differentiated thyroid carcinoma; PTC, papillary thyroid carcinoma.

Univariate analysis identifies the following predictive factors for RAIR disease (adjusted *P*‐value <0.05): mitotic index ≥3/2mm^2^, tumour necrosis, HGDC, and PDTC. Tall cell PTC and other aggressive subtypes of PTC were not statistically associated with the onset of RAIR disease. There was no statistically significant association between *BRAF* mutation and the occurrence of RAIR disease (adjusted *P*‐value = 1.000). Figure 2 illustrates the RAIR‐disease‐free survival curves according to the histological subtypes.

In the multivariable analysis, the mitotic index was no longer associated with RAIR disease. Moreover, after adjustment for tumour necrosis, histological subtypes were no longer statistically associated with RAIR‐free survival. Table 4 presents the multivariable model.

**Table 4:** multivariate model for prediction of RAIR disease evolution including histological type and high‐grade criteria

Variable	Level	HR	Lower 95% CI	Upper 95% CI	Adjusted *p*‐value
Histological type (2022 WHO)	Well‐differentiated carcinomas	1	‐	‐	1.000
	Other aggressive subtype of PTC	1.566	0.181	13.574	
	Tall cell subtype of PTC	1.808	0.482	6.780	
	HGDC	2.590	0.500	13.414	
	PDTC	3.367	0.549	20.662	
Tumour necrosis	None	1	‐	‐	0.009
	Presence	4.272	1.423	12.823	
Mitotic index (/2 mm^2^)	<2	1	‐	‐	1.000
	2–3	1.147	0.349	3.768	
	≥3	1.417	0.430	4.667	

**Figure 3:**
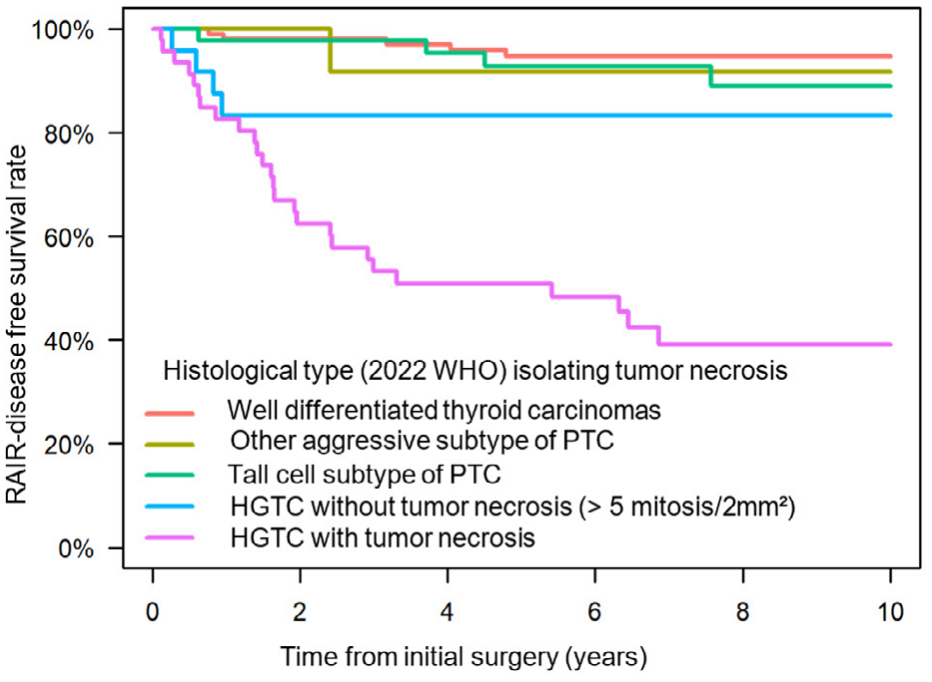
RAIR disease‐free survival curves according to histological type after isolating tumour necrosis.

We therefore performed a new statistical analysis isolating high‐grade carcinomas defined by the presence of tumour necrosis. In this approach, high‐grade carcinomas with necrosis were significantly associated with RAIR disease in univariate analysis, with an HR of 12.742 (95% CI: 6.120–26.531), whereas high‐grade carcinomas defined solely by a high mitotic index were no longer associated with RAIR disease (Figure 3).

### Comparison of Classification Editions

**Figure 4:**
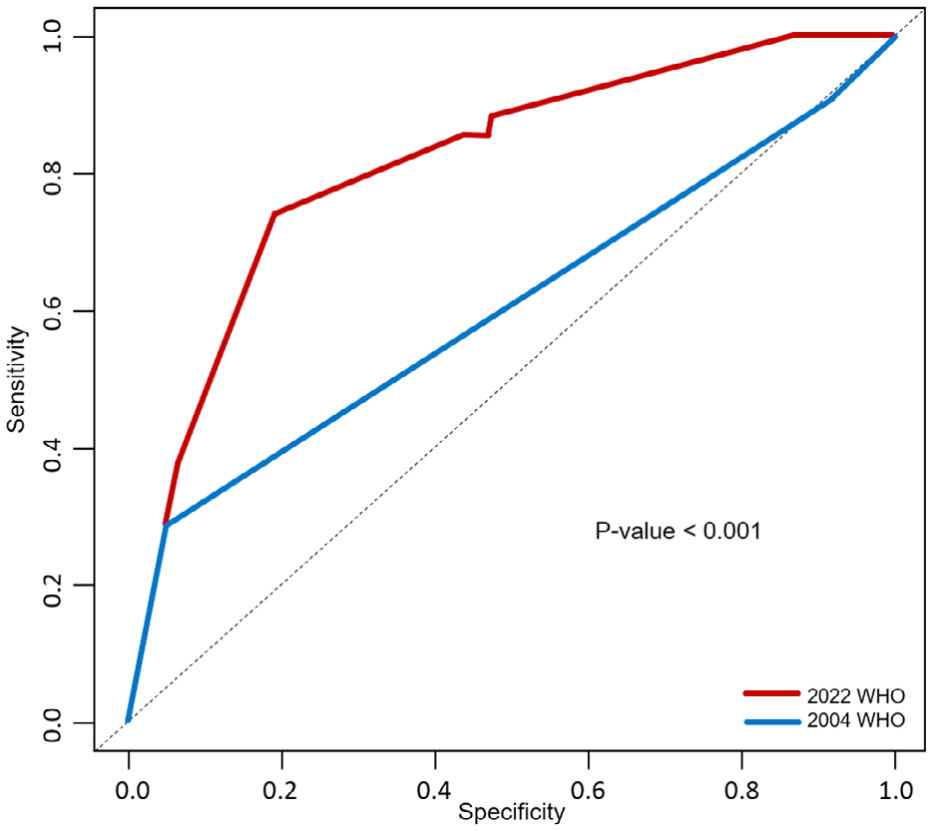
Comparison of WHO classifications (2022 versus 2004) in predicting the onset of RAIR disease.

Figure 4 illustrates the superior performance of the 2022 WHO classification in predicting RAIR disease. The area under curve (AUC) for 2022 WHO was 0.81 (95% CI: 0.74–0.89), compared with 0.60 (95% CI: 0.51–0.69) for 2004 WHO (*P* < 0.001), confirming a significant improvement in predictive value with the 2022 WHO classification.

## Discussion

The objective of this study was to validate the 2022 WHO classification's role in predicting RAIR and identifying predictive histological factors in a cohort of 253 advanced thyroid carcinomas, with a long‐term clinical follow‐up (more than 5 years in 84% of patients).

Upon review, we identified 70 high‐grade carcinomas, including 39 HGDC and 31 PDTC, representing 15% and 12% of the cohort, respectively. These proportions are higher than those reported in the literature, where such lesions represent 1%–6.7% of thyroid carcinomas.^11^ This discrepancy can be attributed to the inclusion criteria of our cohort, which selected larger tumours or those with extrathyroidal extension (pT3, pT4, or M1 stages according to the 2010 pTNM classification). As a result, 40 patients (16%) developed RAIR disease, a proportion higher than the 2%–5% described in previous studies.^12^, ^13^, ^14^ However, it is reported that 50% of high‐grade carcinomas are refractory to iodine,^11^ and our observations are consistent with these data, with 30 cases of RAIR disease (43%) among the 70 high‐grade carcinomas.

Since the introduction of the Turin criteria to define PDTC, studies have suggested that some well‐differentiated carcinomas exhibit a prognosis similar to that of PDTC. As early as 2006, Hiltzik *et al*. proposed a scoring system based on tumour necrosis and the mitotic index to identify these more aggressive tumours despite preserved architecture.^5^ Recent studies, in agreement with our findings, emphasized the importance of high‐grade criteria for a more accurate risk stratification.^11^, ^15^ However, while poorly differentiated carcinoma was already recognized in the 2004 WHO classification, the category of high‐grade carcinomas, including HGDC, was only introduced in the 2022 WHO classification. The current 2022 WHO classification defines high‐grade carcinomas (HGDC and PDTC) as those exhibiting either tumour necrosis or a high mitotic index. The introduction of high‐grade carcinomas in the 2022 WHO classification means that pathologists can no longer rely solely on architectural patterns as a red flag. Instead, they must assess tumour necrosis and the mitotic index in all follicular‐derived carcinomas, irrespective of differentiation or architectural subtype. Our study highlights the importance of these two criteria, associated with RAIR disease in the univariate analysis. However, only necrosis remained significant in the multivariate model. This finding is consistent with that of Rivera *et al*.^12^ who also identified tumour necrosis as an independent prognostic factor for disease‐specific survival, regardless of the mitotic index.

To highlight necrosis as a key prognostic factor for RAIR, we proposed a statistical model separating high‐grade carcinomas with and without necrosis. In this model, high‐grade carcinomas with necrosis were strongly associated with RAIR disease (HR 12.742), whereas those defined solely by a high mitotic index were not. These data suggest that tumour necrosis is a more significant factor than the mitotic index in identifying thyroid carcinomas at risk of progressing to RAIR disease, even though the 2022 WHO classification integrates these criteria within a single category of high‐grade carcinoma.

Moreover, although traditionally considered to have a worse prognosis, aggressive PTC subtypes showed a similar occurrence of RAIR disease to common well‐differentiated carcinomas in our series. These findings contrast with those of a meta‐analysis by Luo *et al*.^13^ which identified tall cell, hobnail, and diffuse sclerosing subtypes as predictive factors for RAIR disease. However, this 2020 study did not consider the high‐grade criteria, which were introduced later in 2022 WHO classification. Futhermore, a more recent study by Ghossein *et al*.^14^ demonstrated that high‐grade PTC is an independent prognostic factor within the tall cell subtype. Their findings suggest that high‐grade features hold greater prognostic value than the histological subtype alone, even when the subtype is traditionally considered aggressive. In addition, this study highlights that high‐grade tall cell PTC is more frequently associated with *TERT* promoter mutations, a known marker of poor prognosis,^16^, ^17^ compared with tall cell PTC without high‐grade features. Similarly, in our study, *TERT* promoter mutation was associated with high‐grade carcinomas in univariate analysis (*P* = 0.003).

In thyroid cancers, the relevance of Ki67 as a prognostic factor is debated.^18^ In our study, Ki 67 was low (≥4 in only 18.2%) and was not statistically associated with RAIR disease in multivariate analysis.

A key development in the 2022 WHO classification is the introduction of the category of low‐risk malignancies, notably NIFTP. NIFTP was already defined in the 2017 WHO classification, and the use of this classification in our cohort would have allowed a more accurate classification of these tumours. Our study identified 28 cases of low‐risk malignancies; all exhibited a benign clinical course, with no recurrence, metastasis, or progression to RAIR disease. These findings are consistent with the literature.^19^, ^20^ In addition, one oncocytic tumour initially classified as PDTC according to the WHO 2004 classification was reclassified as a lesion of UMP after review according to the 2022 WHO criteria. This exceptional reclassification was based on the reinterpretation of necrosis as ischaemic rather than tumour‐related and the absence of unequivocal invasive features, illustrating the stricter diagnostic thresholds in the 2022 classification.

## Conclusion

In conclusion, this study reinforces the significant prognostic value of the 2022 WHO classification and identifies tumour necrosis as a key independent risk factor for RAIR disease. Pathologists should look carefully for high‐grade features in thyroid carcinomas, especially tumour necrosis. Reporting these criteria should become standard pathological practice to decide patient management and follow‐up strategies.

## Author contributions

Conceptualization: Lasolle Hélène, Borson‐Chazot Françoise, and Decaussin‐Petrucci Myriam conceived the study design and research question. Methodology: Lasolle Hélène, Borson‐Chazot Françoise, and Decaussin‐Petrucci Myriam designed the experimental methodology and data collection procedures. Data Collection: André Elise, Mebarki Laurine, Bournaud Claire, Bertholon‐Grégoire Mireille, Descotes Françoise, Lifante Jean‐Christophe were responsible for data collection. Data Analysis: André Elise, Mebarki Laurine, Ilie Mirela, and Subtil Fabien performed statistical analyses and interpreted the data. Writing – Original Draft: André Elise wrote the original draft of the manuscript. Writing – Review and Editing: Lasolle Hélène, Decaussin‐Petrucci, Myriam, Subtil Fabien, Borson‐Chazot Françoise provided critical revisions and editing of the manuscript. Supervision: Lasolle, Hélène, Borson‐Chazot, Françoise, Decaussin‐Petrucci, Myriam supervised the overall project and guided the manuscript development. Funding Acquisition: Lasolle Hélène acquired the funding for the study and managed the budget.

## Conflict of interests

The authors declare no conflicts of interest.

## Funding information

Société Française d'Endocrinologie et Ligue Contre le Cancer.

## Data Availability

The data that support the findings of this study are available from the corresponding author upon reasonable request.
